# Host plant and population source drive diversity of microbial gut communities in two polyphagous insects

**DOI:** 10.1038/s41598-019-39163-9

**Published:** 2019-02-26

**Authors:** Asher G. Jones, Charles J. Mason, Gary W. Felton, Kelli Hoover

**Affiliations:** Department of Entomology, The Pennsylvania State University, University Park, PA 16802 USA

## Abstract

Symbioses between insects and microbes are ubiquitous, but vary greatly in terms of function, transmission mechanism, and location in the insect. Lepidoptera (butterflies and moths) are one of the largest and most economically important insect orders; yet, in many cases, the ecology and functions of their gut microbiomes are unresolved. We used high-throughput sequencing to determine factors that influence gut microbiomes of field-collected fall armyworm (*Spodoptera frugiperda*) and corn earworm (*Helicoverpa zea*). Fall armyworm midgut bacterial communities differed from those of corn earworm collected from the same host plant species at the same site. However, corn earworm bacterial communities differed between collection sites. Subsequent experiments using fall armyworm evaluating the influence of egg source and diet indicated that that host plant had a greater impact on gut communities. We also observed differences between regurgitant (foregut) and midgut bacterial communities of the same insect host, suggesting differential colonization. Our findings indicate that host plant is a major driver shaping gut microbiota, but differences in insect physiology, gut region, and local factors can also contribute to variation in microbiomes. Additional studies are needed to assess the mechanisms that affect variation in insect microbiomes, as well as the ecological implications of this variability in caterpillars.

## Introduction

Insect herbivores inhabit a diverse set of niches, and therefore face a wide variety of challenges such as nutritionally recalcitrant food sources, toxins, environmental extremes, and threats from parasites and pathogens. Insects have integrative strategies to contend with these challenges, which often include forming symbiotic associations with microbes^1^. Microbial associations are ubiquitous among animals, but vary along functional and ecological continua^2^. Compared to endosymbiotic bacteria, the evolutionary trajectories and transmission strategies of facultative gut symbionts are far more variable^3,4^. Extracellular symbionts can be obtained through environmental sources^5^, shared food resources^6,7^, trophallaxis^3^, deposition on egg surfaces, and copulation^7^.

The insect gut can be rich in microbial symbionts, and the associations, locations, and functions of these associates can vary considerably. Some insects possess special gut modifications or structures such as paunches, diverticula, and caeca to house symbionts^8^, while others lack morphological modifications. The roles of insect gut symbionts are diverse. For example, insect gut symbionts can be involved in metabolism of recalcitrant food sources^9,10^, provisioning of vitamins^11^ and nutrients^12,13^, and metabolism of plant allelochemicals^14–16^. Oral bacteria found in the regurgitant can also be involved in manipulating plant responses to herbivore feeding, thereby suppressing induction of plant defences and leading to increases in herbivore growth^17,18^.

Lepidoptera (butterflies and moths) is the third largest insect order with over 200,000 described species. Larval lepidopteran guts are characterised by a simple, tube-like morphology that facilitates the rapid transit of food associated with high consumption rates^19^. These insect guts represent extreme environments for microorganisms due to their high alkalinity (pH > 10)^20–22^.

Commonly, caterpillar gut microbiomes are simple and variable, usually being comprised of relatively few dominant taxa^23–26^, and appear to be shaped in part by dietary and environmental sources^26–28^. Bacterial communities have also been described from lepidopteran eggs^25,29^, suggesting there is potential for maternal transmission of microbiota. The roles of lepidopteran oral and gut bacteria in facilitating plant–insect interactions remain unclear, but they likely have facultative functions, particularly in relation to plant defences^14,30^. The sources of gut microbiota in lepidopterans have received little attention; understanding the factors that influence bacterial community composition may shed light upon symbiont–host co-adaptation and the strategies insects use to acquire their microbial partners.

The lepidopteran family Noctuidae includes many polyphagous agricultural pests that can cause significant economic losses. *Spodoptera frugiperda* (fall armyworm) is a highly polyphagous noctuid that is a major agricultural pest in South America, the Caribbean, and most recently in Africa^31,32^. In the northern United States and Canada, sporadic fall armyworm infestations occur from populations that annually migrate from southern Texas and Florida as this species cannot overwinter in the cooler northern climates^33^. Although fall armyworm develops faster and prefers grasses such as maize and wheat, it can also complete development on broadleaf crops such as soybean and cotton^34^. Corn earworm (*Helicoverpa zea*) is another agricultural noctuid pest that is distributed throughout the American continent^35^. Its major host plant is maize, although it will also feed on other crops such as tomato, cotton and soybean^36^.

Despite the economic importance of corn earworm and fall armyworm, little is known about the composition of their midgut and oral bacteria and how these communities are shaped by transmission from eggs and host plant feeding. We used bacterial 16S-rRNA sequencing to determine if there were differences in gut communities from different population and/or host plant sources. Specifically, our objectives were to: (i) characterise and compare the midgut bacterial community of wild-collected fall armyworm and corn earworm from different locations in Pennsylvania, (ii) determine whether egg source (wild-collected or laboratory-reared) or diet source (soybean leaves or corn silk) were more important in shaping fall armyworm midgut bacterial communities, and (iii) compare bacterial communities present in the regurgitant with those of the midgut in fall armyworm. This latter objective is of interest because specific bacteria from oral secretions (regurgitant) of lepidopterans have been shown to influence plant defences^30^, but whether these bacteria are a subset of those found in the midgut or comprise a unique community is unknown. To address these objectives, we used culture-independent high throughput sequencing methods. We also used traditional culture-based and 16S sequencing methods to examine bacteria present in the regurgitant of fall armyworm collected from Pennsylvania and Puerto Rico.

## Results

### Comparisons of midgut microbiota between fall armyworm and corn earworm

**Figure 1:**
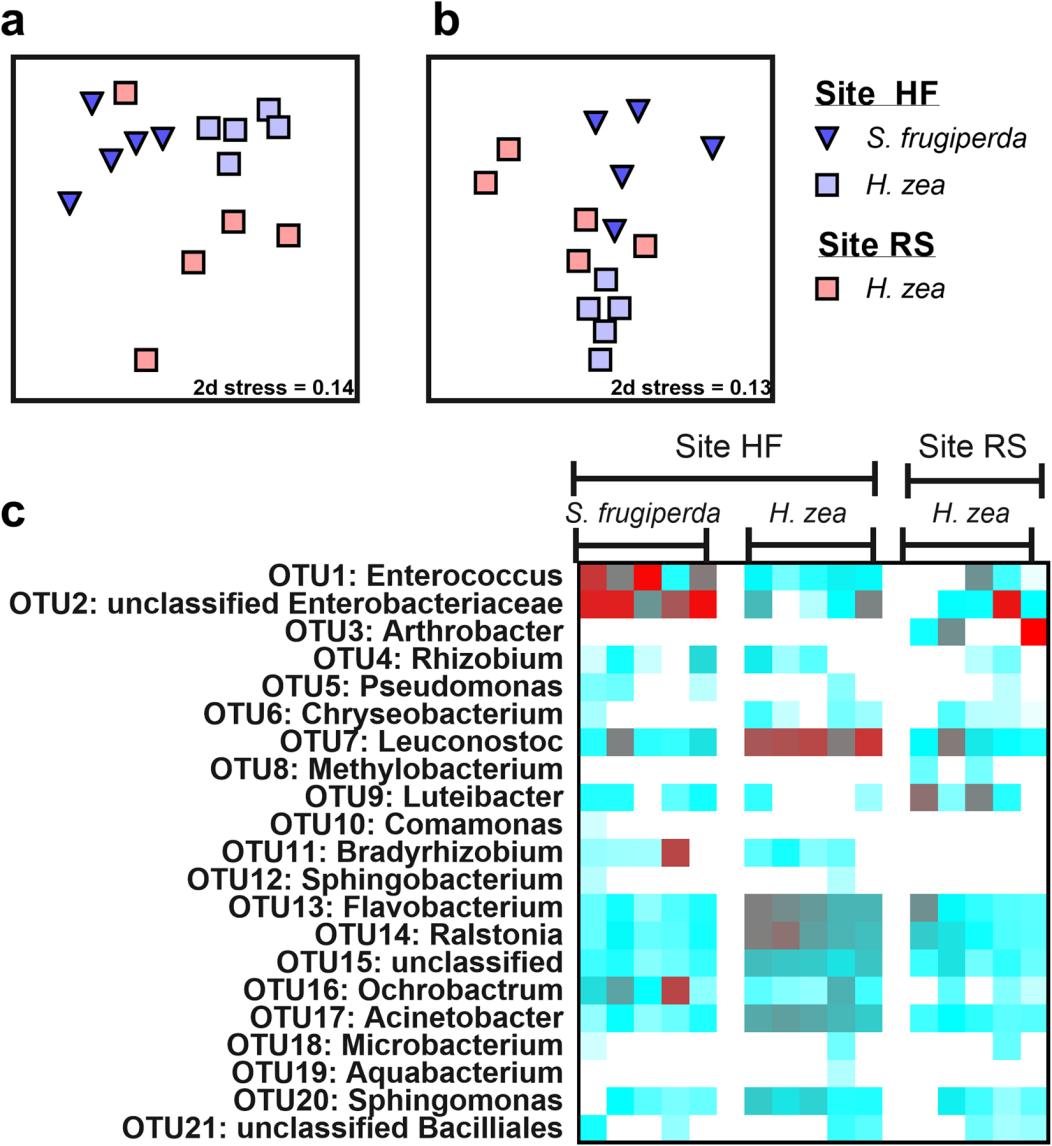
Differences between fall armyworm (*Spodoptera frugiperda*) and corn earworm (*Helicoverpa zea*) midgut bacterial communities collected from sweet corn ears in Pennsylvania. Corn earworm was collected from Site HF (Harner Farm) and Site RS (Rock Springs), while fall armyworm was collected only from Site HF. Non-metric multidimensional scaling (nMDS) plots were constructed using Bray-Curtis (**a**) and Jaccard (**b**) dissimilarities. Stress values indicate a good fit in two dimensions. Heat maps (**c**) show relative abundance (log_2_ transformed) of OTUs (operational taxonomic units). Red boxes indicate greater relative abundances, blue boxes correspond to lower values, and white boxes correspond to no detection of that particular OTU.

There were significant differences in the bacterial communities inhabiting the midguts of fall armyworm and corn earworm (Fig. 1a,b; Supplemental Fig. 1). Multivariate analyses revealed significant differences between fall armyworm and corn earworm microbiota based on Bray-Curtis (Fig. 1a; PerMANOVA p < 0.001) and Jaccard (Fig. 1b; PerMANOVA p < 0.001) similarity indices. In addition to the differences between these two species collected at the same location, corn earworm collected from different locations also exhibited divergent communities (Fig. 1). We found substantial differences in the bacterial midgut communities of corn earworm obtained from the two sites in Pennsylvania, despite the fact they were collected from the same host plant species. Alpha-diversity metrics also differed between lepidopteran species, with fall armyworm exhibiting marginally significantly lower Simpson (p = 0.053) and Shannon (p = 0.033) metrics than corn earworm (Supplemental Fig. 1; Supplemental Table 1). This suggests that corn earworm midgut microbiota exhibited greater levels of diversity and greater evenness.

Heat maps of midgut microbiota indicated that conspecific individuals exhibited a high degree of variability in terms of both membership and abundance (Fig. 1c). Among the most enriched OTUs (operational taxonomic units), fall armyworm had the greatest relative abundances of *Enterococcus* and unclassified Enterobacteriaceae. Corn earworm from the HF population had lower relative abundances of *Enterococcus* and unclassified Enterobacteriaceae, but were enriched for *Leuconostoc*. Corn earworm from the RF site were highly variable as to which OTUs were present in high relative abundances, and had fewer *Leuconostoc* than corn earworm from the HF site.

### Influence of egg population source and plant diet on fall armyworm midgut bacterial communities

**Figure 2:**
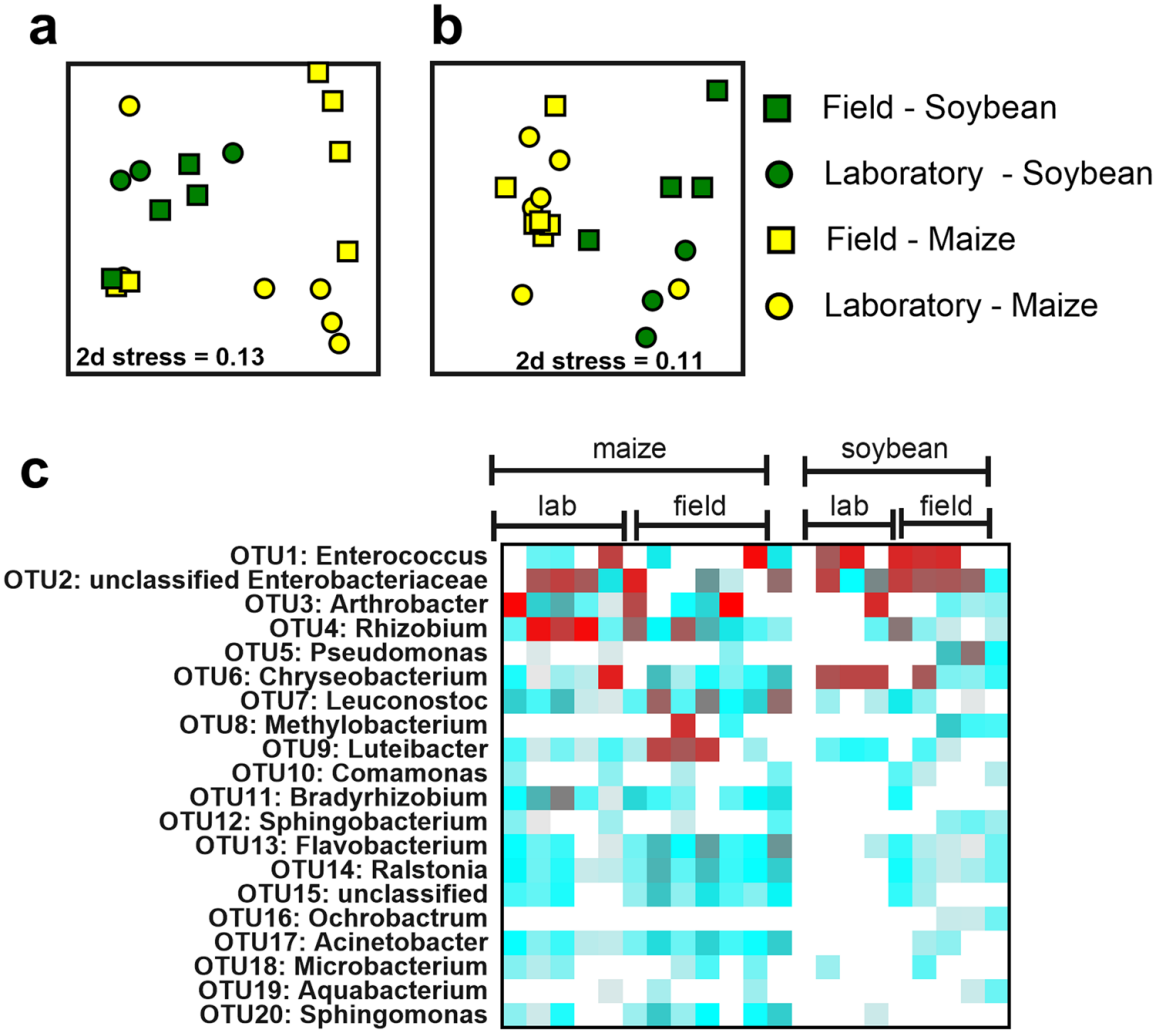
Influence of diet (maize or soybean) and egg population source (field-collected or laboratory) on fall armyworm (*Spodoptera frugiperda*) midgut bacterial communities. Non-metric multidimensional scaling (nMDS) plots were constructed using Bray-Curtis (**a**) and Jaccard (**b**) dissimilarities. Stress values indicate a good fit in two dimensions. Heat maps (**c**) show relative abundance (log_2_ transformed) of OTUs (operational taxonomic units). Red boxes indicate greater relative abundances, blue boxes correspond to lower values, and white boxes correspond to no detection of that particular OTU.

Multivariate analyses revealed there was a significant impact of host plant on midgut bacterial communities in terms of both Bray-Curtis (Fig. 2a) and Jaccard (Fig. 2b) similarities (Table 1). There was no impact of egg population source or its interaction with host plant on midgut bacterial communities (Fig. 2a,b; Table 1). At both the order and individual OTU levels, there was substantial variation in abundance of bacterial taxa between individual fall armyworm larvae within treatments (Fig. 2c; Supplemental Fig. 2). In general larvae feeding on maize had high relative abundances of OTUs corresponding to unclassified Enterobacteriaceae, *Rhizobium*, and *Chryseobacterium*. Soybean-fed larvae generally had higher relative abundances of *Enterococcus*, unclassified Enterobacteriaceae, *Chryseobacterium*, and *Pseudomonas* (Fig. 2c).

**Table 1:** PERMANOVA output assessing differences between fall armyworm (*Spodoptera frugiperda*) midgut bacterial communities between egg population source (field or laboratory) and host plant (maize or soybean).

Source	df	SS	MS	Pseudo-F	p-value
**Bray-Curtis Similarity**					
Plant	1, 19	8057	8057	4.15	**0**.**029**
Population	1, 19	3407	3407	1.75	0.156
Plant*Population	1, 19	1447	1447	0.74	0.512
**Jaccard Similarity**					
Plant	1, 19	8025	8025	2.07	**0**.**002**
Population	1, 19	4381	4381	1.13	0.181
Plant*Population	1, 19	4167	4167	1.07	0.297

PERMANOVAs were generated using 999 permutations, and the individual insect was included in the model as a random effect. Bold values indicate significant (p < 0.05) differences.

Alpha-diversity metrics indicated that, in general, gut bacterial communities of larvae fed on soybean were more diverse than those fed on maize (Supplemental Fig. 3, Supplemental Table 2). Midguts from insects reared from field-collected and laboratory (Benzon) egg sources generally had the same levels of OTU richness. However maize-fed larvae from the field population had higher bacterial gut diversity than those from the laboratory population (Supplemental Fig. 3).

### Differences in fall armyworm bacterial communities in midgut and regurgitant

**Figure 3:**
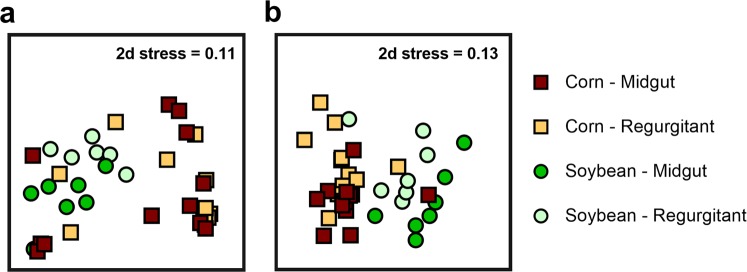
Influence of sample type (midgut or regurgitant) and diet (maize or soybean) on fall armyworm (*Spodoptera frugiperda*) bacterial communities. Non-metric multidimensional scaling (nMDS) plots were constructed using Bray-Curtis (**a**) and Jaccard (**b**) similarities. Stress values indicate a good fit in two dimensions.

We collected paired samples corresponding to fall armyworm regurgitant and midguts to determine if there were differences between the microbiota that reside in the foregut versus the midgut. There were substantial differences in the communities present, and like in prior analyses, there was a major impact of host plant on the midgut bacterial communities using both Bray-Curtis (Fig. 3a) and Jaccard (Fig. 3b) similarities (Table 2). In addition to these differences between host plants, there was a substantial impact of whether the community of origin was from the midgut or regurgitant (Table 2; p = 0.013). However, we observed no interaction between the host plant and the physical origin of the bacterial community. These trends were consistent for both Bray-Curtis and Jaccard metrics.

**Table 2:** PERMANOVA output assessing differences between fall armyworm (*Spodoptera frugiperda*) bacterial communities between sample type (midgut or regurgitant) and host plant (maize or soybean).

Source	df	SS	MS	Pseudo-F	p-value
**Bray-Curtis Similarity**					
Sample type	1, 39	7191	7191	3.82	**0**.**013**
Plant	1, 39	16257	16257	8.64	**0**.**001**
Sample type*Plant	1, 39	2075	2075	1.10	0.331
**Jaccard Similarity**					
Sample type	1, 39	4772	4772	1.22	**0**.**020**
Plant	1, 39	11526	11526	2.95	**0**.**001**
Sample type*Plant	1, 39	4222	4222	1.08	0.169

PERMANOVAs were generated using 999 permutations, and the individual insect was included in the model as a random effect. Bold values indicate significant (p < 0.05) differences.

Using Bray-Curtis similarities, we used a two-way SIMPER analysis to identify the major OTUs that contributed to the differences between host plant and regurgitant, and then conducted paired statistical tests. While host plant had a significant impact on the relative abundance of several of the OTUs that inhabited the midgut and regurgitant (Table 3), the effects of regurgitant were far more limited. Only *Enterococcus* exhibited significant differences between midgut and regurgitant from fall armyworm feeding on maize (p = 0.045) and soybean (p = 0.001).

**Table 3:** Pairwise comparisons of abundances of individual bacterial OTUs (operational taxonomic units) between host plant (soybean or maize) and sample type (midgut or regurgitant) in fall armyworm (*Spodoptera frugiperda*). Bold values indicate significant (p < 0.05) differences. OTUs were selected using a SIMPER analysis.

OTU	Maize		Soybean		Plant		Sample type (maize)		Sample type (soybean)	
	Midgut	Regurgitant	Midgut	Regurgitant	W	p-value	W	p-value	W	p-value
OTU001: *Enterococcus*	27.48 ± 10.8	9.82 ± 6.2	32.94 ± 5.95	15.43 ± 2.69	427	**0**.**0055**	185	**0**.**0449**	98	**0**.**0006**
OTU002: Enterobacteriaceae	39.93 ± 10.9	55.15 ± 9.46	22.25 ± 5.52	27.74 ± 9.76	253	**0**.**0387**	131	0.2913	59	0.3823
OTU003: *Arthrobacter*	5.33 ± 4.66	4.14 ± 3.1	24.49 ± 4.97	34.01 ± 7.78	455	**0**.**0003**	159.5	0.5968	50	0.065
OTU004: *Rhizobium*	6.87 ± 3.44	2.31 ± 0.85	0.91 ± 0.36	1.24 ± 0.63	348	0.5944	143	0.7125	57	0.2786
OTU005: *Pseudomonas*	0.97 ± 0.4	1.44 ± 0.58	0.07 ± 0.03	0.09 ± 0.05	192	**<0**.**0001**	140	0.5899	65	0.798
OTU008: *Methylobacterium*	1.46 ± 0.64	1.73 ± 0.81	0.04 ± 0.02	0.03 ± 0.01	222	**0**.**0028**	155.5	0.7679	65	0.798
OTU011: *Bradyrhizobium*	0.98 ± 0.38	0.91 ± 0.42	0.04 ± 0.03	0.01 ± 0	191	**<0**.**0001**	158	0.6707	61.5	0.5169
OTU015: Unclassified	3.85 ± 2.42	1.25 ± 0.47	0.02 ± 0.01	0.01 ± 0.01	181.5	**<0**.**0001**	157	0.7122	58	0.2821
OTU016: *Ochrobactrum*	1.43 ± 0.79	0.5 ± 0.16	0.76 ± 0.33	1.15 ± 0.61	328	1.000	154	0.8426	59	0.3667
OTU018: *Microbacterium*	0.48 ± 0.25	1.25 ± 0.7	0.56 ± 0.17	0.7 ± 0.31	364.5	0.3207	128	0.2184	62	0.5737
OTU033: *Bacillus*	0.02 ± 0.02	0.29 ± 0.2	2.1 ± 1.56	2.11 ± 1.39	468.5	**<0**.**0001**	116.5	0.3205	63	0.6454

### 16S sequencing of individual isolates from Puerto Rico and Pennsylvania

**Figure 4:**
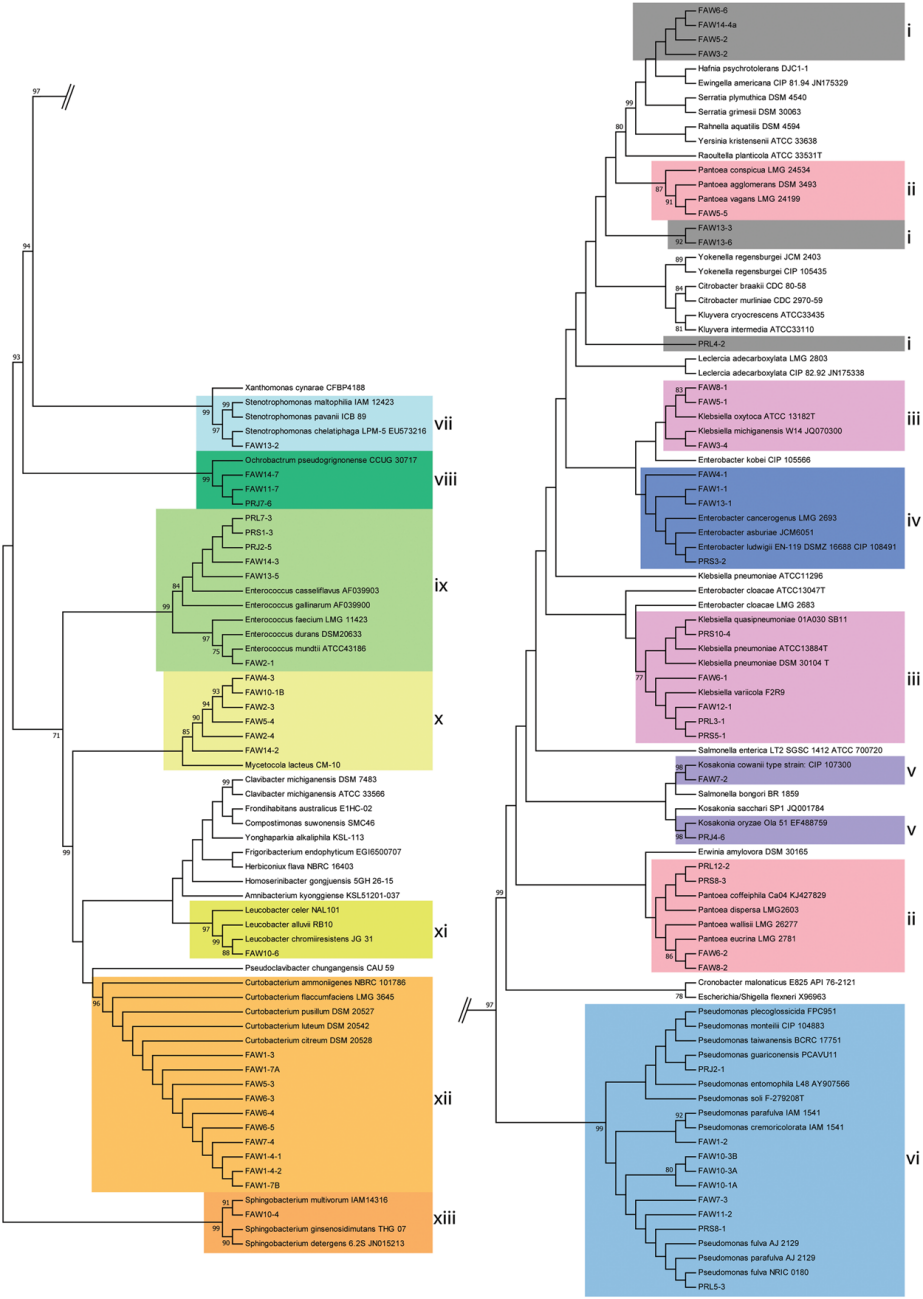
Phylogenetic tree of near full-length 16S rRNA gene sequences from bacterial isolates cultured from fall armyworm (*Spodoptera frugiperda*) regurgitant collected in Pennsylvania, United States (indicated by FAW prefix) Puerto Rico (indicated by PR prefix), and selected type strains from the RDP database. The phylogenetic tree was constructed using the Maximum Likelihood method based on the Jukes-Cantor model in MEGA7. The tree with the highest log likelihood (−5718.26) is shown. Bootstrap values with values of 70 or greater are shown. Annotations correspond to clades that are identified as likely the following genera (i) unresolved (ii) *Pantoea* (iii) *Klebsiella* (iv) *Enterobacter* (v) *Kosakonia* (vi) *Pseudomonas* (vii) *Stenotrophomonas* (viii) *Ochrobactrum* (ix) *Enterococcus* (x) *Mycetocola* (xi) *Leucobacter* (xii) *Curtobacterium* and (xiii) *Sphingobacterium*.

Culture-dependent sequencing of bacteria isolated from the regurgitant of fall armyworm collected in both Puerto Rico and Pennsylvania support the findings of the community analysis data reported above. We found that bacterial taxa in the Enterobacteriaceae, Pseudomonadaceae and Enterococcaceae and Microbacteriaceae were commonly identified based on sequencing of the V4 region of the bacterial 16S gene (Fig. 4). Common bacterial genera that were isolated include *Pantoea* (Fig. 4, clade ii), *Klebsiella* (Fig. 4, clade iii), *Enterobacter* (Fig. 4, clade iv), *Pseudomonas* (Fig. 4, clade v), *Ochrobactrum* (Fig. 4, clade vii), *Enterococcus* (Fig. 4, clade viii), *Mycetocola* (Fig. 4, clade x) and *Curtobacterium* (Fig. 4, clade xi). In some cases, the first six of these genera were detected in larvae collected in both Pennsylvania and Puerto Rico.

## Discussion

Bacterial gut communities of two lepidopteran species, fall armyworm and corn earworm, were variable and influenced by multiple factors. Bacterial communities were distinct depending on insect species and collection location. We also found that host plant was much more important in shaping midgut bacterial communities in fall armyworm than the population source of the eggs. Midgut and regurgitant bacterial composition of fall armyworm were also distinct, suggesting that the physiology of different gut regions may affect bacterial composition or abundance.

We found high variability in gut bacterial composition and abundance between individuals of the same species, even from those feeding on the same food source. This is consistent with reports of several other lepidopteran species that possess microbial gut assemblages that differ between individuals^29,37,38^. The high variability in lepidopteran gut bacterial assemblages and the apparent lack of a resident microbiota has fuelled speculation that gut associates lack functional importance in lepidopterans^38–41^. These suggestions likely stem from the limited research into functional roles of gut bacteria in Lepidoptera. However, demonstrated roles of lepidopteran oral and gut bacteria include suppression^30^ and detoxification^14^ of plant defences. Moreover, these associates can mediate other ecological associations, such as interactions with entomopathogens^42–44^. Future studies manipulating the presence/absence and assemblage of gut bacterial associates in controlled studies are needed to address functions of these associates and the consequences of variations in community composition/abundance.

Although we observed high intraspecific variation in bacterial gut composition and structure, we also found that the two lepidopteran species had distinct midgut bacterial communities despite feeding on the same host plant species at the same location. This suggests that acquisition of bacteria from the environment is not a completely stochastic process and that bacterial communities are not merely a reflection of the host plant microbiome. There are likely gut physiological mechanisms that select for certain taxa, which may help shape bacterial composition in these different species. Competition among taxa may also play a role and it is likely that microbial community composition is dynamic. The ability of insect guts to filter certain taxa from the wider environmental pool of bacteria has been observed in several systems including cockroaches^45^, bean bugs^46–48^ and bumble bees^49^. There is little known about the mechanisms involved in bacterial acquisition and establishment in lepidopteran guts, but variation in physiochemical properties and competition among taxa are likely important for shaping these communities.

Host plant (maize or soybean) played a much bigger role in influencing the composition of midgut bacteria in fall armyworm than egg source (Fig. 2). These results are comparable to studies on other folivores showing that host plant can shape gut bacterial communities, including *Spodoptera littoralis*^24^, *Helicoverpa* spp.^24,26,37^, *Lymantria dispar*^23,28^, and *Leptinotarsa decemlineata*^50^. There are pre- and post-digestive interactions between the plant and microbe that may affect these communities. For example, differences in leaf surface, wax composition, availability of sugars, and interactions with other bacterial species can alter the bacterial community composition present on plant leaves^51^, therefore altering the composition and quantify of bacteria the insect may encounter. There are also chemical interactions occurring in the gut that may mediate these interactions; for example, foliar concentrations of plant secondary compounds can affect bacterial composition in insect guts^52^. To what degree fall armyworm gut bacterial composition reflects that of their host plant was not investigated in this study, but is likely influenced by a combination of phyllosphere bacteria and the chemical composition of the plant tissues.

We also found that corn earworm midgut bacterial communities from two sites in Pennsylvania were distinct, even though the host plant (sweet corn) was the same at both sites, likely due to differences in phyllosphere bacteria inhabiting the host plants at each site. Several studies investigating factors that influence phyllosphere bacteria found that geographical location of plants altered bacterial community composition^53,54^.

Although fall armyworm individuals possessed different overall bacterial communities in paired regurgitant and midgut samples, pairwise comparisons revealed that this was primarily driven by a single *Enterococcus* OTU. There are several physiological and morphological differences between the midgut and foregut that may differentially impact microbial gut associates. The foregut is covered in a cuticular lining while the midgut cells are protected by the peritrophic membrane lining the midgut wall. Additionally, the pH of the lepidopteran alimentary canal changes across its length; the midgut is highly alkaline, while the foregut is usually closer to neutral^55,56^. These differences may provide differential binding and colonization affinities for microbiota, resulting in greater abundances of certain microbial taxa. The foregut may also pose greater disturbances to bacterial communities, because the cuticular lining is replaced during each moult. It is unclear if and how microbes recolonise the foregut after moulting, although in *S*. *littoralis* fluorescently-labelled bacteria were observed in the foregut throughout different instars^57^.

We observed relatively few bacterial taxa associated with the regurgitant of fall armyworm. A recent study of the “regurgitome” of Mexican bean beetle (*Epilachna varivestis*) revealed a highly diverse microbiome with 1230 bacterial species in 577 genera^58^. In our study we observed less diversity and richness; culture-independent sequencing detected about 100 OTUs in larvae fed on maize and soybean. In both plant species, *Enterococcus* and an unclassified Enterobacteriaceae OTU comprised the majority of the taxa in our study. These results paired well with our culture-dependent survey. Culture-dependent 16S sequencing of bacteria from regurgitant of fall armyworm larvae collected in Pennsylvania and Puerto Rico showed that isolates belonged to genera including *Pantoea*, *Enterobacter*, *Klebsiella*, *Pseudomonas*, *Curtobacterium*, *Mycetocola*, and *Enterococcus*. These genera are commonly associated with many different insect groups^59^, and have documented roles in mediating fall armyworm–plant interactions^30^.

Based on the phylogenetic tree constructed from sequencing near-full length 16S-rRNA, we found some bacterial isolates could not be reliably identified (Fig. 4i) and several genera including *Pantoea*, *Klebsiella*, *Enterobacter*, and *Kosakonia* were not monophyletic. The 16S rRNA gene is widely used to study bacterial ecology, but can have limitations distinguishing closely related taxa due to high sequence similarities^60^. The Enterobacteriaceae tends to have poor resolution using hypervariable regions of the 16S rRNA gene, including the broadly used V4 region^61,62^. It is likely that the unclassified Enterobacteriaceae OTU in our culture-independent analyses actually contains several bacterial genera, and thus underestimated the diversity in our samples.

This study contributes to our understanding of the factors that influence gut microbiomes in two lepidopteran insects and offers insight into the composition of bacterial communities in fall armyworm regurgitant, which are likely to be relevant for mediating plant–insect interactions. We show that variation in insect host, gut region, and host plant can affect the composition of bacterial gut communities of lepidopteran larvae. Further research will be required to identify the factors that alter bacterial communities at each of these levels of scale.

## Methods

### Insect and plant sources

Plant and insect material were collected from two sites in Centre County, PA in September 2016: a local farm in State College, PA (HF) and the Pennsylvania State University Russell E. Larson Research Farm (RS). These two sites are separated by approximately 11 km (Supplemental Table 3). We obtained fall armyworm from HF, and corn earworm from HF and RS. Insects were collected from the ears of sweet corn (*Zea mays*). At the time of insect collection, we also collected soybean (*Glycine max*) foliage and maize silk and stored them at 4 °C. Fall armyworm from HF were used for culture-based sequencing of bacteria in regurgitant and bacterial community analysis of regurgitant and midguts. Corn earworm collected from HF and RS were used for bacterial community analysis of midguts.

Fall armyworm larvae were also collected from three sites in southern Puerto Rico in February 2017, which included two local farms (PRL and PRS) and the Juana Diez Experimental Research Station (PRJ) (Supplemental Table 3). Fall armyworm from the Puerto Rico sites were used for culture-based analysis of regurgitant. Upon removal from the field, larvae were placed in individual plastic cups with maize silk from the plant they were collected from for food, until sample collection. For larvae from Site HF and RS, regurgitant and midgut samples were collected the following day. For larvae collected in Puerto Rico, due to shipping time back to the United States, regurgitant was collected three days after larval collection.

A laboratory colony of fall armyworm was obtained from Benzon Research (Carlisle, PA), and maintained at Pennsylvania State University. To determine the contributions of host plant and egg source on gut bacterial composition in fall armyworm we used egg masses produced by 1) the Benzon laboratory colony and 2) field-collected fall armyworm. The field-collected eggs were produced by the moths that developed from caterpillars originally collected from Site HF on September 7, 2016. These caterpillars were final instars when collected and were fed exclusively on maize silk collected from the same site until they pupated, with the aim of maintaining their natural gut microbiota. Upon hatching, neonate larvae were placed in individual cups with either soybean leaves (FS Hisoy HS33A14-98SB132B) or maize silk (var. Providence), from plant material collected from Site RS. Larvae were fed *ad libitum* and leaves were replaced every 2–3 days until larvae reached the final instar. Regurgitant and midgut collections were conducted on the second day of the final instar.

### Culture-based sequencing of regurgitant bacteria

Regurgitant was collected by gently squeezing larvae with soft forceps until they regurgitated. The regurgitation droplet was collected directly from the caterpillar’s oral cavity using a sterile pipette tip^30^. One µl of regurgitant was diluted with sterile Milli-Q water. Serial dilutions were plated on 2xYT media and the number of colony-forming units (CFUs) per ml of regurgitant was quantified. Colonies that displayed unique morphology were subcultured and these pure cultures were stored as glycerol stocks at −80 °C. Pure cultures were prepared from glycerol stocks on solid media for 48 h. DNA was extracted using the CTAB protocol^63^ and PCR was performed on extracted DNA using the 16S primers 27F (5′ AGAGTTTGATYMTGGCTCAG 3′) and 1392R (5′ ACGGGCGGTGTGTRC 3′) with GoTaq® Green Master Mix (Promega, Madison, WI, USA). The PCR conditions were as follows: initial denaturation at 95 °C for 2 min; followed by 30 cycles of 95 °C for 1 min, 50 °C for 45 s, 72 °C for 1 min; then 72 °C for 5 min. PCR products were purified using Exo-SAP-it (Affymetrix. Santa Clara, CA) following manufacturer’s instructions. Sanger Sequencing of purified DNA products was performed at the Penn State Genomics Core Facility, University Park, PA.

Contigs for bacterial sequences were assembled and trimmed using SeqMan Pro (DNASTAR, Madison, WI). Consensus sequences were used to search the Ribosomal Database Project database for bacterial type strains with similar (0.80–1.00 match) sequences^64,65^. The Muscle algorithm within MEGA7 software^66^ was used to align the bacterial type strains and samples. The aligned sequences were then used to construct an unrooted phylogenetic tree using the Maximum Likelihood method based on the Jukes-Cantor model^67^ in MEGA7.

### Sample collection and DNA extraction

Regurgitant was collected from larvae as described above. Approximately 20 µl of regurgitant was collected from each larva and stored at −80 °C until needed. After regurgitant collection, caterpillars were starved for 2–3 h, surface sterilised in 10% Coverage Plus NPD (Steris, Mentor, OH, USA), and rinsed twice in sterile molecular grade water. Midguts were dissected under sterile conditions and stored at −80 °C until DNA extraction. DNA extractions were performed using the Quick-DNA™ Fecal/Soil Microbe Microprep Kit (Zymo Research, Irvine, CA, USA) according to manufacturer’s instructions.

### Generation and sequencing of 16S amplicons

Primers used for bacterial V4 16S-rRNA amplification were 515F and 806R^68^. Amplicons were generated in 25 μL volumes using Phusion Hi-Fidelity Polymerase (New England BioLabs, Ipswich, MA, USA) containing 0.5 μM of forward and reverse primers and 25 ng of template DNA. Reaction conditions for 16S amplification were: 94 °C 3 min, 30 cycles of 94 °C for 45 sec, 50 °C for 60 sec, and 72 °C for 90 sec, followed by a final extension of 72 °C for 10 min. Indices and Illumina sequencing adapters were added to amplicon pools with 5 additional cycles of PCR. Barcoded products were pooled and sequenced on an Illumina MiSeq using 2 × 300 bp paired end reads. Generation of amplicon pools and sequencing of products was completed by the PSU Hershey Genomics Facility.

### Processing of sequencing data

Bacterial amplicons were processed and analyzed using mothur v. 1.37^69^. The recommended workflow was modified such that the mothur command ‘pcr.seqs’ was implemented with pdiffs = 2 after the command ‘make.contigs’ to remove primer sequences from the reads. Bacterial operational taxonomic units (OTUs) were picked at 97% similarity and used for subsequent 3000 analyses. We used mothur to conduct OTU subsampling to OTUs. Taxonomies were determined using a mothur-formatted version of the Ribosomal Database Project (v. 9). Prior to subsequent analysis, samples were evaluated against a negative sequencing control, and two OTUs were removed from subsequent analyses.

### Statistical analyses

Analyses of the bacterial communities were conducted using non-metric multidimensional scaling (nMDS) ordination and permutation-based multivariate analysis of variance (PerMANOVA). Heat maps were generated using the 20 most abundant OTUs by performing a log_2_ [x] transformation. We used Bray-Curtis similarities generated from standardised data that incorporated relative abundance of OTUs to assess community structure; Jaccard similarities were generated using subsampled data incorporating presence/absence to assess composition. PerMANOVA and nMDS was conducted in PRIMER-E (v. 7.0) using these metrics. PERMANOVA was also run in PRIMER-E using 999 iterations of the model. First, we assessed if there were differences between corn earworm and fall armyworm collected from the field using a one-way analysis. Then, we evaluated if insect egg source and plant sources have impacts on fall armyworm gut communities using a two-way analysis. Finally, we determined if there were differences between gut and regurgitant communities, and if there was an impact of host plant using a two-way analysis. Following this last test, we used similarity percentages (SIMPER) analysis to identify the OTUs that drive the differences, and then conducted non-parametric Wilcoxon rank sum analysis on the relative abundances.

## Supplementary information

Supplementary Material

## Data Availability

The datasets generated during and/or analysed during the current study are available from the corresponding author on reasonable request. Short read sequences have been submitted to NCBI SRA under accession number PRJNA507591. Sanger sequences of individual bacterial isolates have been submitted to NCBI Genbank Under Submission Number SUB4856027.
