# Supplementary material for: Host plant and population source drive diversity of microbial gut communities in two polyphagous insects
Source: Sci Rep. 2019 Feb 26;9:2792. doi: 10.1038/s41598-019-39163-9 (PMC6391413; doi:10.1038/s41598-019-39163-9)
Supplement: Supplementary file 1 — Supplementary Material [file 41598_2019_39163_MOESM1_ESM.pdf]

Host plant and population source drive diversity of microbial gut communities in two polyphagous insects

Asher G. Jones[1*], Charles J. Mason[1], Gary W. Felton[1], Kelli Hoover[1]

[1]Department of Entomology, The Pennsylvania State University, University Park, PA 16802, USA

Supplementary Material

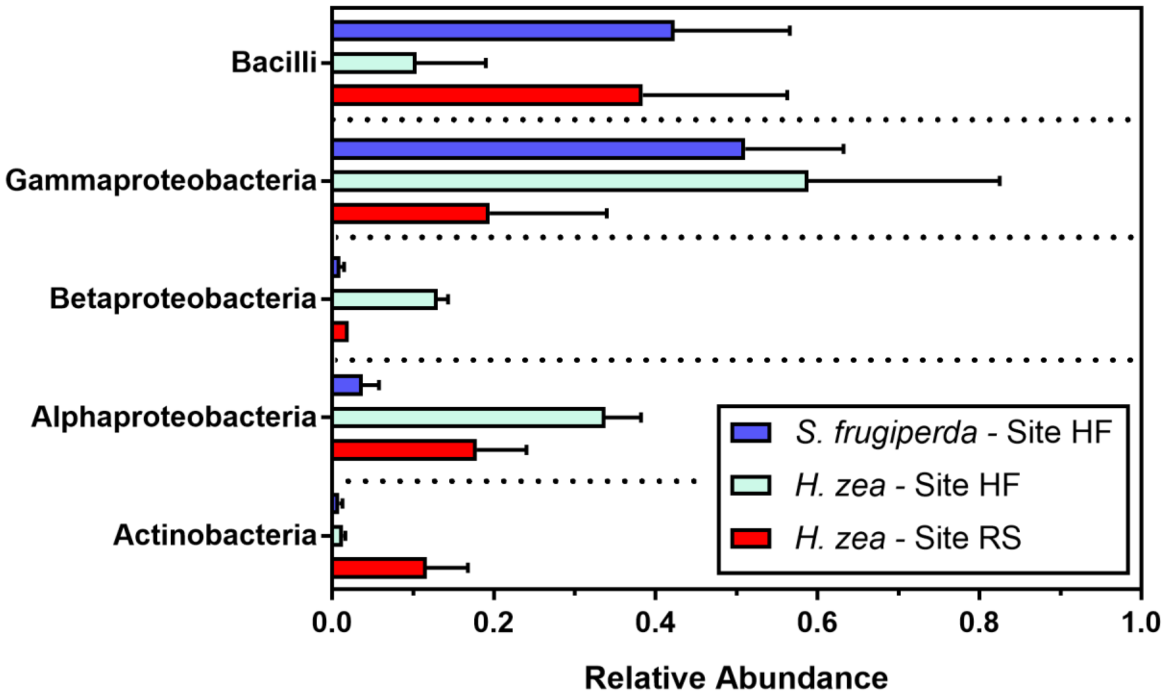

Supplemental Figure 1: Relative abundance of the most common bacterial orders present in fall armyworm (*Spodoptera frugiperda*) and corn earworm (*Helicoverpa* zea) midguts (> 95% of the abundances). Bars represent means with standard errors.

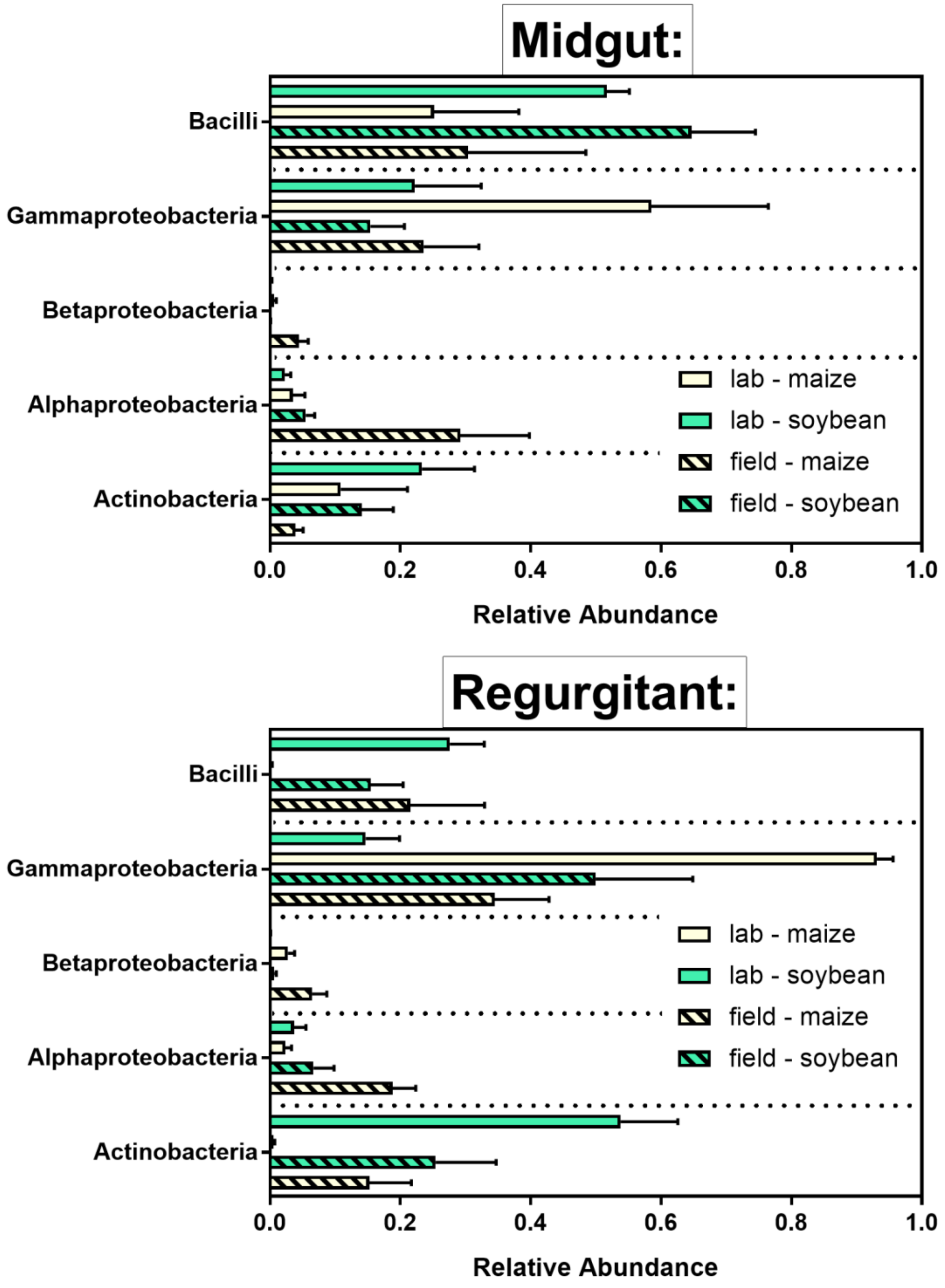

Supplemental Figure 2: Relative abundance of the most common bacterial orders present in fall armyworm midguts (top) and regurgitant (bottom) (> 95% of the abundances). Different colours represent different host plants (soybean or maize). Open bars indicate lab populations; hatched bars indicate field populations. Bars represent means with standard errors.

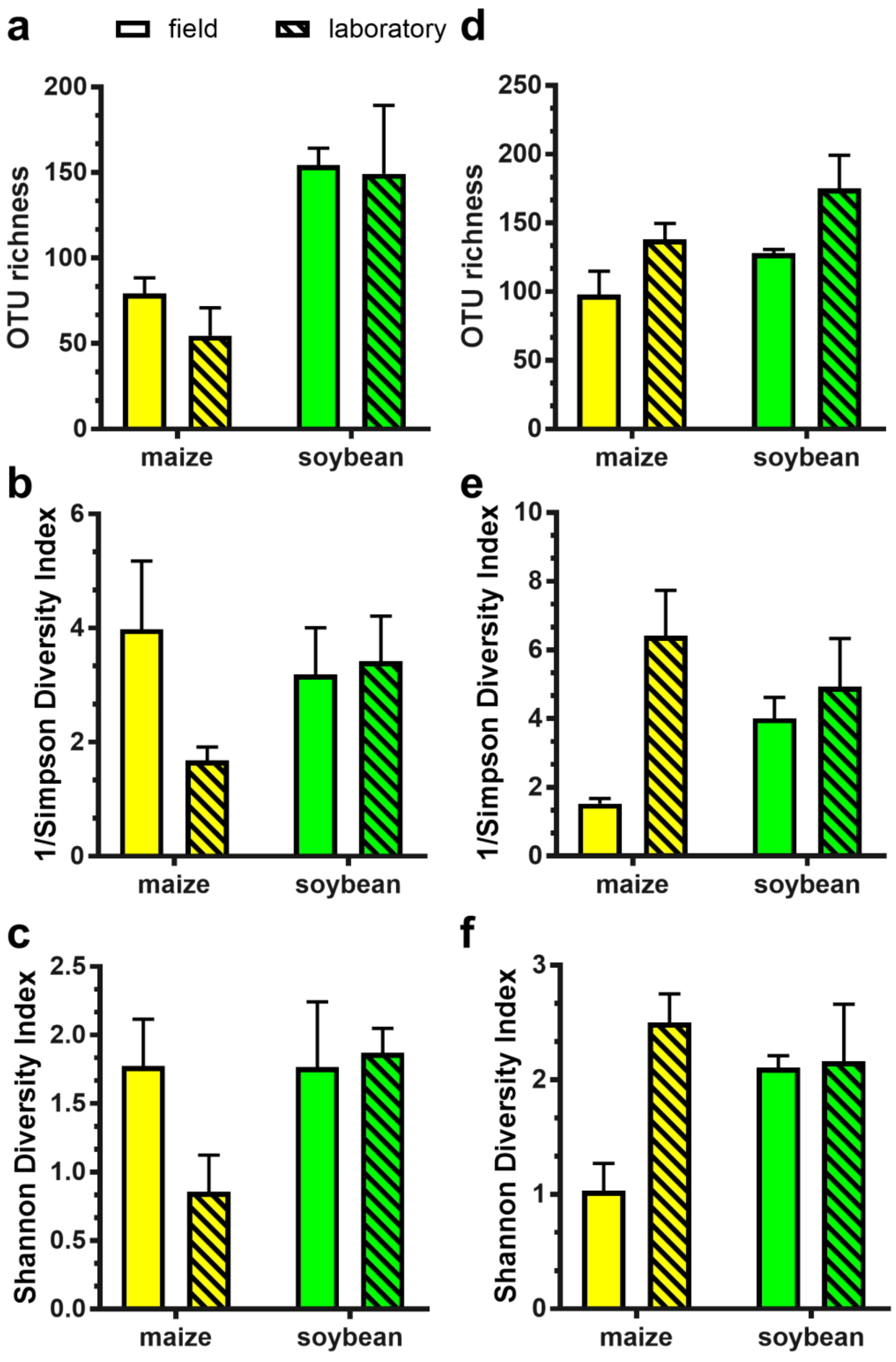

Supplemental Figure 3: Alpha diversity indices of midgut (a-c) and regurgitant (d-f) bacterial communities in fall armyworm (*Spodoptera frugiperda*).

Supplemental Table 1: Comparisons between alpha diversity of field-collected fall armyworm (*Spodoptera frugiperda*) and corn earworm (*Helicoverpa zea*) Numbers represent means (std. errors).

	S. frugiperda	*H. zea*	t-value	p-value
OTUs	85.4 (10.6)	190.2 (77.5)	1.15	0.281
1 / Simpson	2.23 (0.2)	10.7 (6.2)	2.26	0.053
Shannon	1.4 (0.2)	2.7 (0.8)	2.45	0.033

Supplemental Table 2: ANOVA of midgut and regurgitant alpha diversity in fall armyworm (*Spodoptera frugiperda*)

		Plant		Egg source		Interaction	
		F-value	P-value	F-value	P-value	F-value	P-value
Midgut :	OTUs	8.83	**0.009**	1.59	0.226	0.107	0.748
	1 / Simpson	1.25	0.282	3.97	0.065	2.3	0.15
	Shannon	2.45	0.155	2.98	0.105	1.87	0.192
Regurgitant:	OTUs	4.73	**0.053**	6.23	**0.025**	0.032	0.86
	1 / Simpson	0.26	0.642	11.2	**0.004**	3.46	0.083
	Shannon	1.09	0.313	11.5	**0.004**	5.93	**0.028**

Supplemental Table 3: Site descriptions and locations of field-collected plant and insect material

Site	Location	Coordinates	Crops grown	Material collected	Date collected
HF	Centre County, PA	40.7601319<br>-77.8787313	Sweet corn	*H. zea* larvae<br>*S. frugiperda* larvae	3-7 Sep 2016
RS	Centre County, PA	40.7106224<br>-77.9644764	Soybean<br>Sweet corn	*H. zea* larvae<br>Soybean foliage<br>Corn silk	7 Sep 2016
PRL	Lajas, PR	18.03161111<br>-67.07361111	Field corn	*S. frugiperda* larvae	14-15 Feb 2017
PRS	Salinas, PR	17.96122222<br>-66.26805556	Sorghum	*S. frugiperda* larvae	14-15 Feb 2017
PRJ	Juan Diez, PR	18.02763889<br>-66.52722222	Field corn	*S. frugiperda* larvae	14-15 Feb 2017
